# Kinetic and optical properties of a new probe for sulfatase activity assay

**DOI:** 10.1016/j.dib.2017.04.056

**Published:** 2017-05-05

**Authors:** Hey Young Yoon, Jong-In Hong

**Affiliations:** Department of Chemistry, College of Natural Sciences, Seoul National University, Seoul 08826, Republic of Korea

## Abstract

A new probe for detecting sulfatase activity generated fluorescent *N*-methylisoindole when it was treated with sulfatase. Detailed synthetic procedures of reaction intermediates are described along with their spectral data. The Diels-Alder adduct of *N*-methylisoindole and *N*-phenylmaleimide was identified by LC–MS data. The fluorescence changes and kinetic values of the probe upon sulfatase treatment in the presence and absence of reducing agents were described. Inhibitory potency of EMATE was measured using the probe in the presence and absence of reducing agents. In addition, the fluorescence intensities of the probe upon sulfatase treatment were also monitored at pH 5 and 7 with and without reducing agents.

**Specifications Table**

**Table:** 

Subject area	*Biological Chemistry*
More specific subject area	*enzymatic assays and inhibitor screening*
Type of data	*text file, graph*
How data was acquired	*UV/Vis spectrometry, NMR, mass spectrometry, microplate reader.*
Data format	*Raw and analyzed*
Experimental factors	*Before measurement of inhibitory potency, pre-incubation with EMATE and sulfatase was carried out at 37* *°C overnight.*
Experimental features	*Fluorescence measurements of the probe with sulfatase under various conditions were performed*
Data source location	
Data accessibility	*Within this article*

**Value of the data**

• The synthesis procedure will provide researchers with a general preparation method of sulfatase substrates.
• The kinetic parameters of the probe and inhibitory potency showed the effects of reducing agents.
• The fluorescence properties at pH 7 and pH 5 showed the utility of the probe under neutral conditions.

## 1 Data

A new probe to detect sulfatase activity was developed. When the probe was treated with sulfatase, it generated fluorescent N-methylisoindole which was influenced by reducing agents and pH conditions [1]. The fluorescence intensity, kinetic parameters and inhibitory potency under Tris buffer and reducing agents were described. Fluorescence intensities of the probe with sulfatase at pH 5 and 7 were compared.

## 2 Experimental design, materials and methods

### 2.1 Chemical synthesis

^1^H and ^13^C NMR spectra were collected on a Bruker Advance DPX-300. Fast atom bombardment mass spectrometry (FAB-MS) data were obtained using a JEOL JMS-AX505WA mass spectrometer with m-nitrobenzyl alcohol (NBA) as a matrix and the data were reported in units of mass to charge (*m/z*). Analytical thin layer chromatography was carried out using Kieselgel 60F-254 plates purchased from Merck and column chromatography was performed on Merck silica gel 60 (70–230 mesh). All chemical reagents purchased from either Sigma-Aldrich or TCI and used without any further purification.

Probe **1** was synthesized following previously reported procedures [2], [3], [4], [5], [6].



A solution of sulfuryl chloride (1.62 ml, 20 mmol) in Et_2_O was cooled to −75 °C under nitrogen. A solution of neopentyl alcohol (1.76 g, 1 equiv.) and pyridine (1.62 ml, 1 equiv.) in Et_2_O was added dropwise to the cooled sulfuryl chloride solution for 1 h and stirred at room temperature for 2 h. The white precipitates were removed by filtration and the filtrate was concentrated in vacuo. The product was used for the next synthesis step without further purification.

**Compound 7**: To a solution of 4-hydroxybenzyl alcohol (1.7 g, 1 equiv.) in anhydrous THF was sodium hydride (437 mg, 1.1 equiv.) slowly added at 0 °C. After 10 min, crude neopentyl chlorosulfate was added to the solution at 0 °C and the reaction mixture was allowed to warm up to room temperature and stirring was continued overnight. Upon completion of the reaction, water was added to the reaction mixture to quench sodium hydride and THF was removed in vacuo. Ethyl acetate was added to the residue and the organic layer was washed with brine, then dried over Na_2_SO_4_ and concentrated in vacuo. The crude product was purified by silica gel chromatography (chloroform:acetone=50:1) to give compound **7** (970 mg, 17% yield). ^1^H NMR (300 MHz, CDCl_3_) δ 1.02 (9H, s), 4.10 (2H, s), 4.74 (2H, s), 7.31 (2H, d, *J*=8.7 Hz), 7.44 (2H, d, *J*=8.5 Hz).

**Compound 8**: Pyridine (257 μl, 1 equiv.) was added to a solution of 4-nitrophenyl chloroformate (640 mg, 1.1 equiv.) in anhydrous THF at 0 °C and the reaction mixture was stirred for 20 min. Then, compound **7** (970 mg, 3.54 mmol) dissolved in anhydrous THF was added dropwise within 10 min, and the mixture was stirred at room temperature for 16 h. After the reaction was complete, THF was removed in vacuo, the residue were dissolved in ethyl acetate. The resulting solution was washed with saturated aqueous NH_4_Cl solution several times and concentrated in vacuo. The crude product was purified by silica gel column chromatography (chloroform:acetone=100:1) to yield compound **8** (784 mg, 1.78 mmol, 50% yield). ^1^H NMR (300 MHz, CDCl_3_) δ 1.03 (9H, s), 4.13 (2H, s), 5.32 (2H, s), 7.38 (2H, d, *J*=8.5 Hz), 7.41 (2H, d, *J*=9.0 Hz), 7.52 (2H, d, *J*=8.6 Hz), 8.30 (2H, d, *J*=8.5 Hz); ^13^C NMR (75 MHz, CDCl_3_) δ 25.86, 31.90, 69.75, 83.69, 115.331, 121.43, 121.81, 125.28, 125.46, 130.30, 133.64, 145.39, 150.48, 155.45; HRMS (FAB): *m/z* calcd. for [C_19_H_21_NO_9_S+Na^+^] 462.0835, found 462.0838.

### 2.2 Fluorescence changes upon addition of various concentrations of reducing agents

Sulfatases from *Helix Pomatia* (S9626) were purchased from Sigma-Aldrich. UV/vis spectra were collected on a Beckman DU-800 and fluorescence spectra were measured on a Jasco FP-6500 and SpectraMax M2 spectrophotometer.

**Fig. 1:**
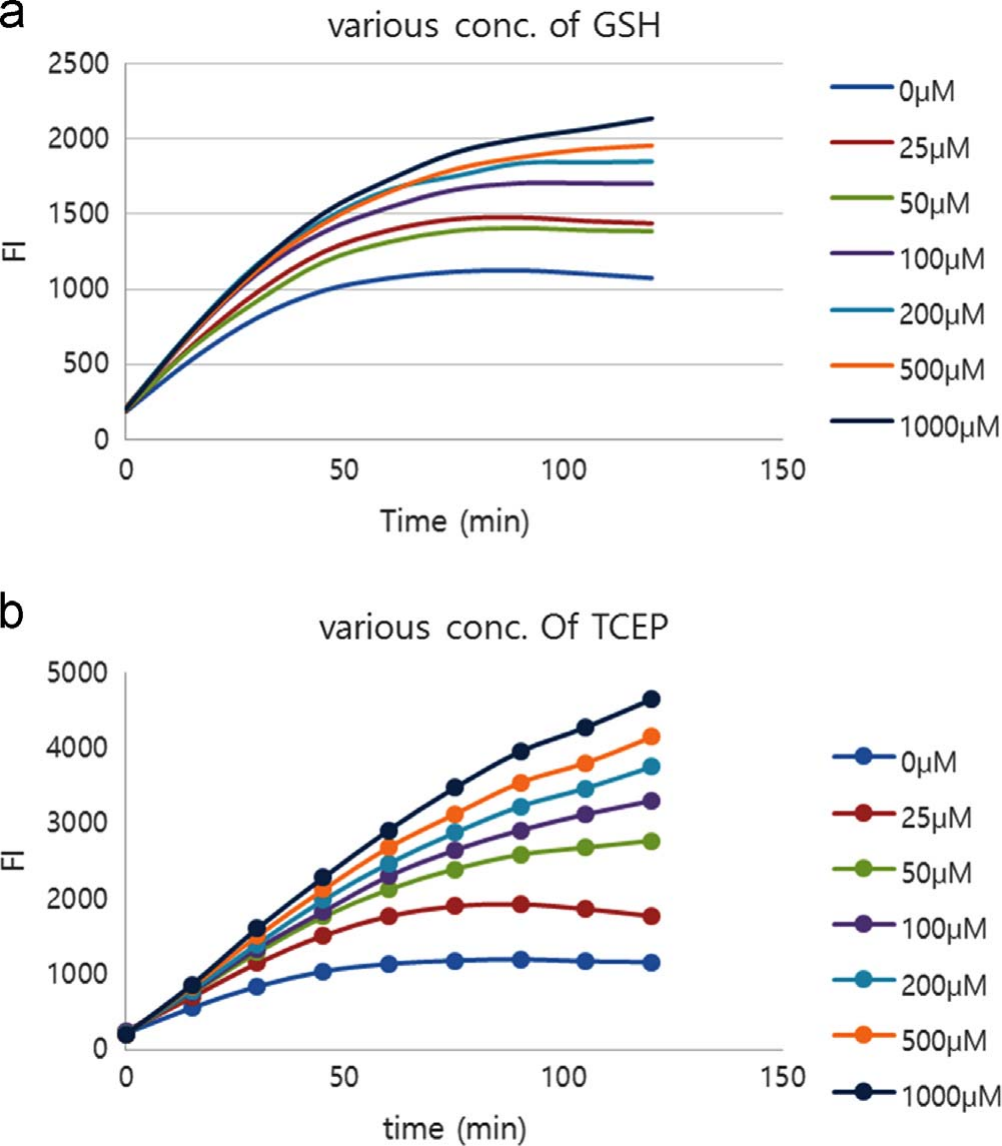
The time-course (0–120 min) fluorescence intensity changes of probe 1 (20 μM) and sulfatase (0.5 mg/ml) upon addition of various concentrations (0, 25, 50, 100, 200, 500, 1000 μM) of (a) GSH and (b) TCEP.

Fluorescence intensities of 20 μM probe upon treatment of 0.25 mg/ml sulfatase in 50 mM Tris buffer in the presence of various concentrations (0, 0.025, 0.05, 0.1, 0.2, 0.5 and 1 mM) of GSH or TCEP were monitored in time-dependent manner (Fig. 1).

### 2.3 Diels-Alder reactions and LC/MS data

**Fig. 2:**
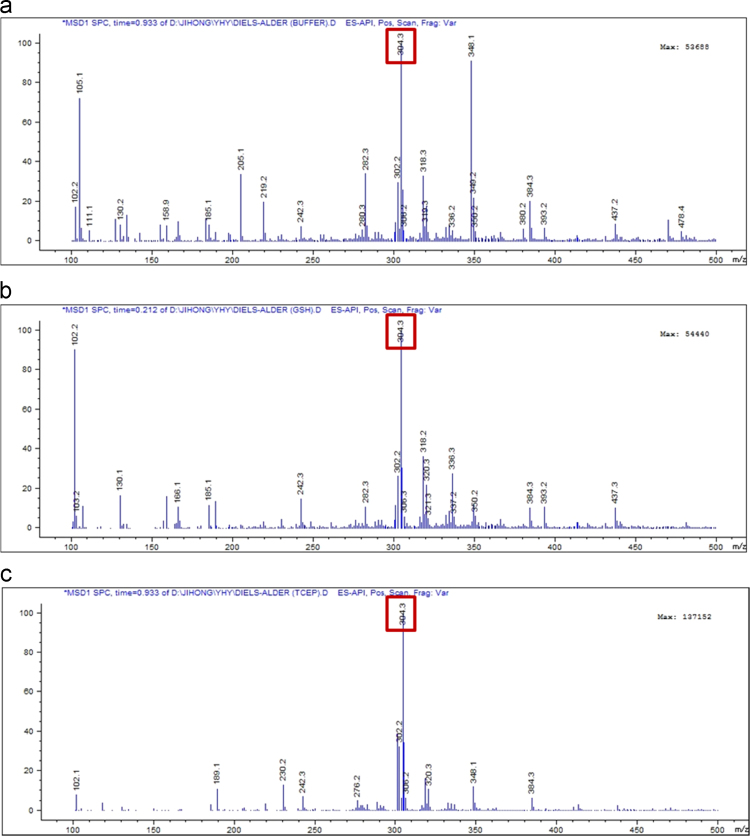
Mass analysis after Diels-Alder reactions between *N*-phenylmaleimide and products of probe 1 with sulfatase in 50 mM Tris buffer containing (a) no additives, (b) 1 mM GSH, and (c) 1 mM TCEP.

A mixture of 0.25 mg/ml sulfatase and 20 μM probe **1** in 50 mM Tris buffer was incubated for 45 min to generate *N*-methylisoindole and then, the mixture was poured into *N*-phenylmaleimide solution in THF and stirred at 70 °C overnight. The reaction mixture was extracted with CH_2_Cl_2_, dried over Na_2_SO_4_, filtered, and concentrated *in vacuo*. The residue was dissolved in 1 mL of methanol and subjected to liquid chromatography–mass spectrometry (LC/MS) analysis (Agilent technologies 1260 infinity) (Fig. 2) to monitor the formation of Diels-Alder adducts.

The same procedures were carried out in 1 mM GSH-containing buffer or 1 mM TCEP-containing buffer.



### 2.4 Determination of kinetic parameters

**Fig. 3:**
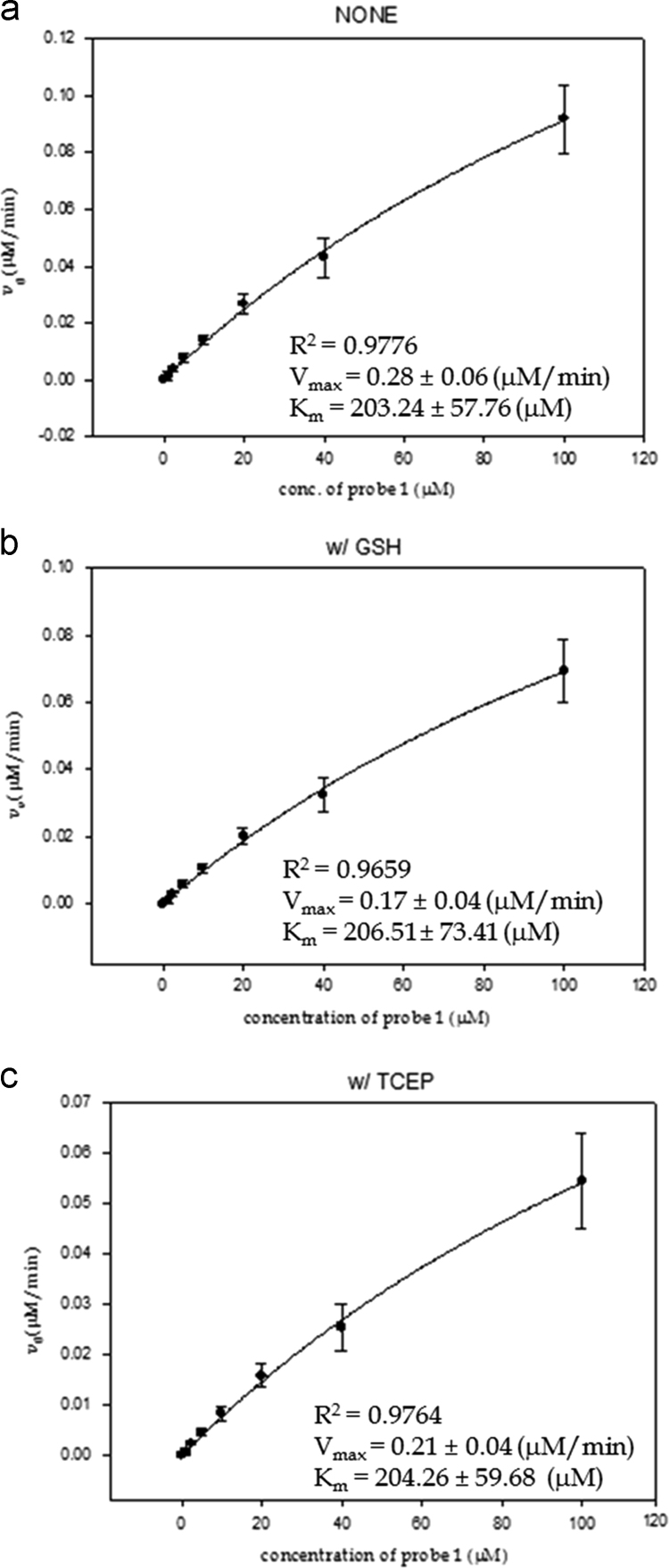
Calculated *K*_m_ and *V*_max_ values when probe 1 was reacted with sulfatase in 50 mM Tris buffer containing (a) no additive, (b) 1 mM GSH, and (c) 1 mM TCEP.

To measure kinetic parameters, 0.25 mg/mL sulfatase and various concentrations of probe **1** (50, 20, 10, 5, 2.4, 1.2 μM) in 50 mM Tris buffers with and without 1 mM reducing agents (pH 7.4, 37 °C) were used. The rate of fluorescence enhancement at 415 nm when excited at 325 nm was measured to determine the kinetic values (*K*_m_ and *V*_max_) which were calculated by nonlinear fitting of the Michaelis–Menten equation using SigmaPlot 8.0 (Systat Software Inc.) (Fig. 3).

### 2.5 Determination of inhibitory potency

Probe **1** was added to a mixture of sulfatase and estrone 3-O-sulfamate (EMATE) kept at 37 °C overnight. To monitor the fluorescence changes, 0.25 mg/ml sulfatase, 20 μM probe **1** and various concentration of EMATE (0, 12, 25, 50, 100, 200 and 500 nM) in 50 mM Tris buffer and 1 mM GSH- or 1 mM TCEP-containing buffer were used (pH 7.4).

**Fig. 4:**
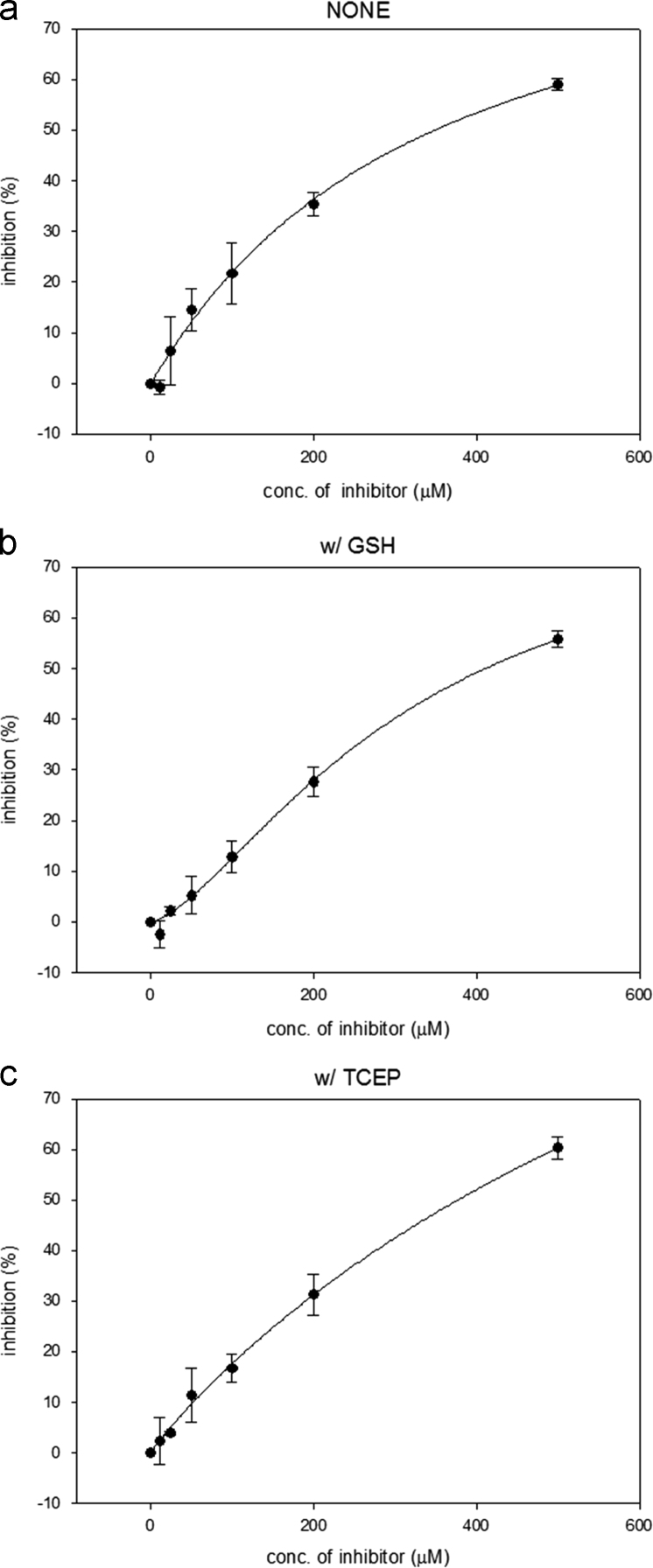
Inhibitory potency when the reaction was carried out in 50 mM Tris buffer containing (a) no additive, (b) 1 mM GSH, and (c) 1 mM TCEP.

The rate of decrease in fluorescence intensity at 415 nm was measured to determine the IC_50_ of EMATE. The IC_50_ values were calculated by fitting with the Hill equation using Sigmaplot 8.0 (Fig. 4).

### 2.6 Fluorescence changes upon sulfatase treatment in acidic conditions

**Fig. 5:**
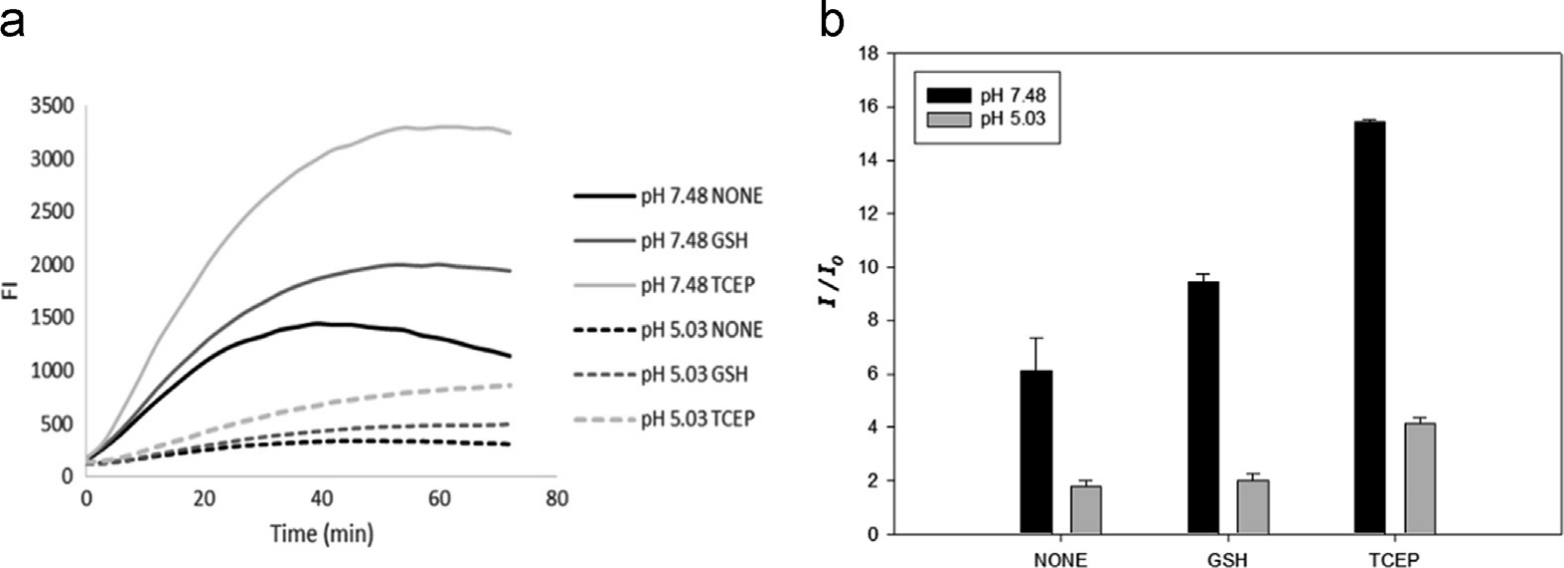
(a) Fluorescence enhancement of probe 1 upon the treatment of sulfatase in neutral and acidic conditions with and without reducing agents in time-dependent manner. (b) Relative fluorescence intensity of probe 1 after 30 min incubation with sulfatase in neutral and acidic conditions with and without reducing agents.

Fluorescence changes of probe **1** with the treatment of sulfatase were measured by treating 20 μM probe **1** with 0.25 mg/mL sulfatase at 37 °C in 50 mM Tris-buffer (100 mM NaCl, 1 mM MgCl_2_, 1 mM CaCl_2_, pH 7.4) and 100 mM potassium acetate buffer (100 mM NaCl, 0.25 mM MgCl_2_, 0.25 mM CaCl_2_, 0.25 mM MnCl_2_ pH 5.0) in the presence and absence of 1 mM GSH or 1 mM TCEP over a period of 0–70 min. Sulfatase from *Helix pomatia* (S9626) was reported to show activity at pH 5.0 and 37 °C [7], [8] (Fig. 5).
